# Visual short-term memory for coherent motion in video game players: evidence from a memory-masking paradigm

**DOI:** 10.1038/s41598-019-42593-0

**Published:** 2019-04-15

**Authors:** Andrea Pavan, Martine Hobaek, Steven P. Blurton, Adriano Contillo, Filippo Ghin, Mark W. Greenlee

**Affiliations:** 1 School of Psychology, University of Lincoln, Brayford Wharf East, LN5 7AY Lincoln, United Kingdom; 2 Department of Psychology, University of Copenhagen, Øster Farimagsgade 2A, DK-1353 Copenhagen, Denmark; 3 University of Ferrara, Dipartimento di Fisica e Scienze della Terra, Via Saragat 1, 44122 Ferrara, Italy; 4 Institute for Experimental Psychology, University of Regensburg, Universitaeststrasse 31, 93053 Regensburg, Germany

## Abstract

In this study, we investigated visual short-term memory for coherent motion in action video game players (AVGPs), non-action video game players (NAVGPs), and non-gamers (control group: CONs). Participants performed a visual memory-masking paradigm previously used with macaque monkeys and humans. In particular, we tested whether video game players form a more robust visual short-term memory trace for coherent moving stimuli during the encoding phase, and whether such memory traces are less affected by an intervening masking stimulus presented 0.2 s after the offset of the to-be-remembered sample. The results showed that task performance of all groups was affected by the masking stimulus, but video game players were affected to a lesser extent than controls. Modelling of performance values and reaction times revealed that video game players have a lower guessing rate than CONs, and higher drift rates than CONs, indicative of more efficient perceptual decisions. These results suggest that video game players exhibit a more robust VSTM trace for moving objects and this trace is less prone to external interference.

## Introduction

There is extensive behavioural evidence that playing action video games enhances a range of perceptual^1–4^ and cognitive functions^5–10^. In addition, it has been demonstrated that training on action video games improves reading abilities in children with developmental dyslexia^11–14^, although this conclusion has been recently disputed^15^. These beneficial effects on cognitive functions may depend on the fact that action video game players have to track, store in visual short-term memory and react to multiple auditory and visual stimuli, which are changing continuously over space and time^3,16^. This massive information processing involves a high degree of perceptual, attentional and motor load (see Karimpur and Hamburger^17^ for a review on the role of action video games in psychological research and Bediou *et al*.^18^ for a meta-analysis on the effects of action video game playing on perceptual and cognitive abilities).

In the present study, we investigated visual short-term memory (VSTM) for parafoveal moving objects in action video game players (AVGPs), non-action video game players (NAVGPs), and non-gaming controls (CONs). The ability to store and maintain task-relevant visual information is crucial to deal with a changing external environment by controlling gaze during visual search^19^, for learning and problem solving^20^, for reducing distraction by maintaining the prioritization of relevant visual information^21^. In general, VSTM reflects the ability of the brain to briefly retain information that can be used to drive learning and/or guide on-going actions^22^. Given its implication in maintaining relevant information from the visual environment, it is important to assess whether it is possible to enhance VSTM with specific training regimes. Such an enhancement is also important because of working-memory limitations in terms of capacity and resources^23–25^. Video game training could provide a cost-effective and reliable tool to improve VSTM, which would be useful for example to counteract normal visual working memory decline in aging^26,27^. Previous studies have shown that AVGPs exhibit superior performance in a change-detection task when compared to persons who do not play video games^28^. Furthermore, AVGPs perform better than non-gamers in a colour-wheel task aimed at measuring VSTM precision^29^, use a more efficient search strategy in a scene change detection task^30^, and perform better on an enumeration task than non-gamers^31^. All these results suggest enhanced VSTM for AVGPs compared to non-gamers. Additionally, Blacker and Curby^20^ using a colour-change detection task showed better performance for AVGPs than NAVGPs, but the VSTM advantage did not depend on the processing speed of the to-be-remembered information. However, Appelbaum *et al*.^32^ showed that AVGPs advantage in perceptual and cognitive tasks may depend on an enhanced sensitivity to visual stimuli, rather than higher capacity or longer retention of information in short-term memory, partially contradicting previous findings.

In this study we further explored whether video game players exhibit better VSTM performance compared to non-gamers in a motion direction change discrimination task. Additionally, we also investigated whether video game players form a visual short-term memory representation that is more robust and less prone to external interference than in non-gamers. Data were also fitted with computational models of VSTM performance and reaction times, the results of which can inform us with respect to the underlying mechanisms in terms of precision, precision variability across trials, guessing rate, stimulus encoding efficiency and response execution.

In particular, we investigated VSTM for moving objects using a memory-masking paradigm similar to that used by Pasternak and Zaksas^33^ in macaque monkeys and by Pavan *et al*.^34^ in humans. There is psychophysical and brain-imaging evidence that VSTM is selective for different attributes of the stimulus including contrast, colour, spatial frequency, orientation, speed and motion direction^35–38^. Pavan *et al*.^34^ investigated VSTM for global motion using a memory-masking paradigm^39^. These authors^34^ presented global motion random dot kinematograms (RDKs) in two visual quadrants, whereas in the two remaining quadrants they presented random-motion RDKs. This pattern of stimulation was displayed in two distinct temporal intervals (i.e., sample and test intervals), separated in time by a 3.2 s delay interval. During the delay interval, directional masking RDKs were presented for 0.2 s in each of the four visual quadrants either at the beginning, in the middle or at the end of the delay interval. The task was to report which RDK in the test interval changed motion direction with respect to the sample interval (direction change discrimination task). The results showed that the mask mainly interfered with performance when displayed 200 ms after the offset of the sample interval, and when its direction, speed and spatial position matched that of the remembered sample (see Pasternak and Zaksas^33^ for similar results on macaque monkeys). These results suggest that the short-term representation for global motion is selective for direction, speed and spatial position, being compromised by intervening directional stimuli presented immediately after the encoding phase^38^. Using a similar paradigm, we investigated whether action video game players encode and retain the direction of coherent and translationally moving stimuli more efficiently than non-gamers. Moreover, we also assessed whether in action video game players, the visual short-term memory trace for coherent motion is more robust and less prone to interference during the retention period. We also wanted to identify the importance of the type of video games engaged in by our participants. To this end we defined a further group of video gamers who engage in non-action video game playing.

Based on earlier studies^20,40^ we predict that action video game players can encode visual motion information more efficiently than their non-playing counterparts and that their visual short-term memory trace is more resistant to interference immediately after the encoding phase.

To test these hypotheses, we used a memory masking discrimination task^33,34^. In the memory interval we presented four RDKs (one in each visual quadrant), two RDKs were composed of random motion, whereas the other two patches were coherent RDKs moving in different directions. After a retention interval of 3.2 s, we presented a test interval, identical to the sample interval with the exception that one of the coherently moving RDK patches changed direction. The task of the participants was to discriminate the location of the moving patch with a different motion direction with respect to the sample. On some of the trials, an intervening masking stimulus was presented 0.2 s after the offset of the sample stimuli. The RDKs composing the masking stimuli were all composed of 100% coherent moving dots with different directions. The temporal parameters of the experiment were chosen on the basis of our previous work on visual short-term memory, in which we found that memory masking is more effective when the mask (1) is presented 0.2 s after the offset of the sample stimuli, (2) it contains directional motion rather than random motion, and (3) its direction of motion and speed match that of the target sample^34^. Masking RDKs contained directional motion since we showed that masking RDKs with random motion did not affect performance when using a similar memory-masking task^34^. It is important to note that, prior to the main experiment, action video game players (AVGPs), non-action video game players (NAVGPs) and non-gamers (CONs) were trained on a direction discrimination task to match their performance. In this fashion, we could be sure that the three groups did not differ with respect to the visual-processing requirements of the tasks.

To anticipate the main findings, the results showed similar VSTM performance in direction change discrimination in all three groups. However, video game players overall outperformed controls showing more accurate VSTM across all the masking conditions. Compared to controls, video game players exhibited less interference in the masking conditions.

## Methods

### Participants

A group of fifteen participants with no previous experience in video game playing (i.e., non-gaming controls: CONs), a group of twelve action video game players (AVGPs), and a group of ten non-action video game players (NAVGPs) took part voluntarily in the experiment. Subjects were matched on age and socio-economic status. The AVGP group was composed of one female and eleven males, the NAVGP group was composed by six females and four males, whereas the CON group was composed by ten females and six males.

In order to be considered an action video game player, the participant needed to have played a minimum of 3 to 4 days a week in the past 6 months and for a minimum of 2 hours a day. On average, AVGPs played action video games 4.75 days a week (SEM = 0.41) and for 5.1 hours a day (SEM = 0.45), whereas NAVGPs played non-action video games (including strategy and role-play games) 4.2 days a week (SEM = 0.63) and for 2.7 hours a day (SEM = 0.49). Independent-sample t-tests revealed a significant difference between the two video-game groups in terms of gaming hours a day (*t*_20_ = 3.708, *p* = 0.001) but not gaming days (*t*_20_ = 0.76, *p* = 0.457).

Action video games played by our observers were, for example, *Call of Duty*, *Battlefield*, *Fallout*, *Far Cry*, whereas non-action video games were for example, *Sims*, *Titan Quest*, *Path of Exile*, *Risen*. There were no obvious effect on the VSTM performance based on the type of action video games played. For the control group, non-gamers had played no video games in the past 6 months. To allocate participants into control and VGP groups, we used the questionnaire adapted from Rosser *et al*.^41^. The questionnaire is useful to determine the genres of the games played and make sure that VGPs played either action or non-action video games for a minimum of 2 hours a day and had not recently played any games from other genres. This is to make sure that video game players were specifically experienced in either action or non-action video games. All participants had normal or corrected to normal visual acuity and sat in a dark room at a distance of 57 cm from the screen. Viewing was binocular.

### Ethical considerations and informed consent

Methods were carried out in accordance with the World Medical Association Declaration of Helsinki^42^. This study was approved by the Ethics Committee of the University of Lincoln (Proposal number: PSY1718534) and the University of Regensburg (Proposal Number: 13-101-0029). Written informed consent was obtained from each participant prior to the enrolment in the study. At the University of Lincoln participants received university credits for their participation, whereas at the University of Regensburg participants were paid for their time.

### Apparatus

Data from ten AVGPs and ten CONs were gathered from the University of Lincoln using a 24-inch Dell P2414H monitor with a refresh rate of 60 Hz and a resolution of 1920 × 1080 pixels. Each pixel subtended 1.66 arcmin. Data from two AVGPs, six CONs and a new sample of ten NAVGPs were collected at the University of Regensburg using a 24-inch Dell S2417DG monitor with a refresh rate of 120 Hz and a resolution of 2560 × 1440 pixels. Each pixel subtended 1.26 arcmin. Though at the University of Regensburg data were collected using a different setup, spatial and temporal parameters were adjusted to determine the same stimulus size and dots’ drifting speed. Stimuli were generated with Matlab Psychtoolbox^43,44^.

### Stimuli

Stimuli were random dot kinematograms (RDKs) consisting of 100 white dots (diameter: 0.15 deg) presented within a circular aperture with a diameter of approximately 8 deg (density: 2 dots/deg^2^). All the dots moved along translational trajectories with 100% coherence. The dots’ Weber contrast was set at 0.99 and moved on a grey background (luminance: 38.2 cd/m^2^) at a speed of 13.3 deg/s. Dots had a limited lifetime; after 83 ms, each dot vanished and was replaced by a new dot at a different randomly selected position within the circular window. Dots also appeared asynchronously on the display^45,46^. In addition, moving dots that travelled outside the circular window were also replaced by a new dot at a different randomly location within the circular window, thus always maintaining the same density. The duration of the motion sequence was 0.2 s.

### Procedure

**Figure 1:**
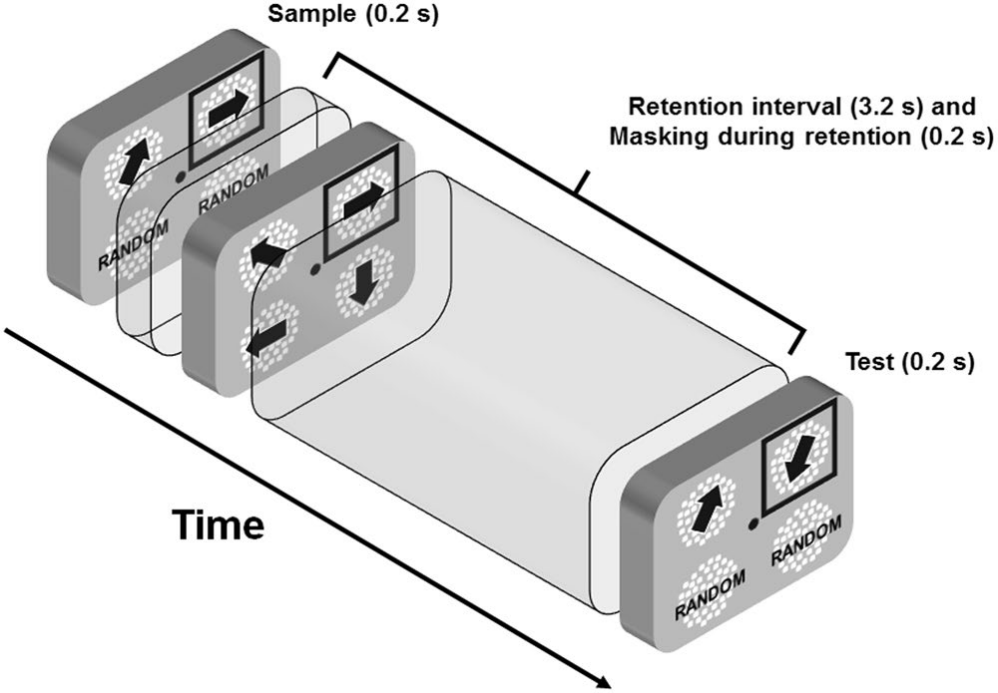
Representation of the procedure used in the memory-masking experiment. The black square frame in the sample interval (not presented during the experiment) indicates the target RDK. In the masking interval, the RDK occupying the same spatial location to that of the target RDK has the same motion direction to that of the target RDK. In the test interval, the same RDK changes direction with respect to the target RDK. The task of the observers was to report which 100% coherently moving patch in the test interval changed direction with respect to the sample interval. They were instructed to ignore the randomly moving RDKs.

The procedure used in the experiment was similar to that reported in Pavan *et al*.^34^ and consisted of two phases: (i) in a control experiment, VGPs and CONs were trained in a motion-direction discrimination task in order to match their performance. This phase of the experiment consisted of a single presentation interval (duration 0.2 s) in which an RDK was displayed in each visual quadrant. One RDK was composed by coherently moving dots (100% coherence), whereas the other three RDKs were random-motion RKDs in which dots were randomly placed within the circular window at every frame (i.e., random position^47^). Participants had to discriminate the motion direction of the coherent RDK which could move in one of the eight cardinal directions (8AFC). Each block consisted of 40 trials, and participants performed as many blocks as needed to get an accuracy equal or higher than 0.95, which was achieved in all cases. (ii) Main VSTM experiment. The sample interval (0.2 s) was composed by two directional RDKs (100% coherence) and two noisy RDKs. Observers were required to remember the location of the two coherent RDKs and their motion directions. A masking interval (duration 0.2 s) was then presented 0.2 s after the offset of the sample stimulus, i.e., 0.2 s after the onset of the retention interval (Fig. 1). The masking interval was composed by four coherently moving patches (100% coherence) with the same speed as the RDKs presented in the sample interval. On each trial, one of the two coherent RDKs that made up the sample was selected as target RDK. In the masking interval, the RDK occupying the same location of the target RDK could drift in the same or different direction with respect to the target RDK (the minimum difference in motion direction between sample and masking was 45 deg). All the other RDKs in the masking interval drifted in different directions than the sample RDKs, but always in one of the eight cardinal directions. After the masking interval there was a delay interval of 3 s in which only the fixation point was presented. It should be noted that the masking interval was presented for 0.2 s during the retention interval, therefore the total duration of the retention interval was 3.2 s (see Fig. 1). After the delay interval we presented a test interval of 0.2 s. In the test interval, the spatial location of the 100% coherent RDKs and the noise RDKs was the same as in the sample interval. However, the RDK occupying the spatial location of the target RDK had a different motion direction, whereas the other RDK had the same motion direction to that of the sample interval. The observers’ task was to judge in which quadrant the direction of the test RDK differed from that of the sample RDK. Coherently moving RDKs could be presented in the upper left and upper right quadrants, upper left and lower left quadrants, lower left and lower right quadrants, upper right and lower right quadrants. Figure 1, for example, represents a trial in which the test RDK presented in the upper right visual quadrant differed from the direction of the sample RDK. Observers were also instructed to ignore the masking interval and only attend to the sample and test RDKs. They responded by pressing one of 4 buttons to indicate the quadrant in which the sample and test RDKs had different directions. Observers performed a total of 288 trials split in 12 blocks (i.e., 24 trials per block). There were 32 trials with no masking. Each of the eight directions of the target sample was repeated 32 times. Moreover, in the masking trials, the direction of the masking RDK presented in the same location to that of the target sample could drift in one of the eight cardinal directions. For example, if the target sample drifted at 90°, the masking patch presented in the same location to that of the target could drift either at 0°, 45°, 90° (sample-mask direction match), 135°, 180°, 225°, 270°, or 315°.

## Results

### Accuracy

The results of the training in motion direction discrimination showed that AVGPs, NAVGPs and CONs needed respectively 2.25 sessions (SEM: 0.58), 2.2 sessions (SEM: 0.2), and 4.87 sessions (SEM: 1.16) to get the desired level of accuracy (>0.95 correct performance rate). A Shapiro-Wilk normality test showed that number of sessions were not normally distributed for the three groups (*p* < 0.05). Additionally, a mean-based Levene test showed that variances were not homogeneous (*p* = 0.002). Therefore, to analyse whether there was a significant difference on the number of training sessions between the three groups, we used an independent-sample Kruskal-Wallis test. The Kruskal-Wallis test did reveal a barely significant effect of group (*χ*^2^ = 6.127, *df* = 2, *p* = 0.047). Bonferroni-Holm corrected Mann-Whitney post-hoc comparisons did not report a significant difference between AVGPs and NAVGPs (*p* = 0.254), between AVGPs and CONs (*p* = 0.123), and between NAVGPs and CONs (*p* = 0.16). However, a non-parametric trend analysis (Jonckheere-Terpstra) showed a significant trend for increasing number of training sessions for the order AVGPs, NAVGPs and CONs (one-sided *p* = 0.0052), suggesting a significant increment of the number of training sessions across the so-ordered groups.

We also compared the performance of the three groups in motion-direction discrimination in the last training session. A one-way ANOVA indicated no significant difference in motion direction discrimination between the three groups post training (*F*_2,34_ = 0.153, *p* = 0.859). Additionally, Bonferroni corrected one-sample t-tests showed that accuracies of AVGPs, NAVGPs and CONs were significantly higher than 0.95 (AVGPs: mean = 0.987, SEM = 0.0049, *t*_11_ = 7.71, *p* = 0.0001; NAVGPs: mean = 0.99, SEM = 0.01, *t*_9_ = 4.00, *p* = 0.003; CONs: mean = 0.985, SEM = 0.00476, *t*_14_ = 7.36, *p* = 0.0001), suggesting equally high performance in all three groups.

Additionally, we tested whether the absolute direction of the target had any effect on performance. A mixed ANOVA on the last training session including as a between-subjects factor the group (i.e., AVGPs, NAVGPs, and CONs) and as a within-subjects factor the target direction (i.e., eight cardinal directions) did not reveal any significant main effect (group: *F*_2,34_ = 0.153, *p* = 0.859, η_p_^2^ = 0.009; target direction: *F*_7,238_ = 0.811, *p* = 0.58, η_p_^2^ = 0.023) or interaction (*F*_14,238_ = 0.7, *p* = 0.775, η_p_^2^ = 0.039), suggesting that at the end of training performance was constant over all target directions.

**Figure 2:**
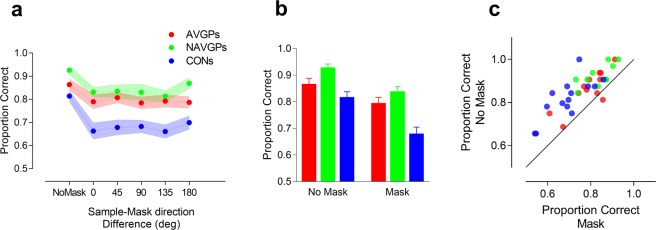
Results of the memory-masking experiment. (**a)** Proportion of correct responses as a function of the sample-mask direction difference (deg) (AVGPs: n = 12, NAVGPs: n = 10, CONs: n = 15). Confidence regions (denoted by colour shading) represent standard error of the mean (SEM). (**b**) Proportion of correct responses for the no-mask and mask conditions. Data were pooled across all sample-mask motion direction differences. Error bars ± SEM. (**c)** Data points represent accuracies (proportion correct) for individual members of the three groups. Mask data points are the mean performance levels for each participant, averaged over each of the five sample-mask direction differences. The black continuous line represents the equal-performance line for mask and no-mask conditions.

We next determined the effect of memory masking on the retention of direction-specific motion information. Figure 2a shows the proportion of correct responses as a function of the direction difference between sample and mask in the memory-masking paradigm used in the main experiment. A mixed ANOVA including as a between-subjects factor the group (i.e., AVGPs, NAVGPs, CONs) and as a within-subjects factor the masking condition (including the no-mask condition and the five direction differences between sample and mask), revealed a significant effect of the group (*F*_2,34_ = 10.1, *p* < 0.001, η_p_^2^ = 0.372), and a significant effect of the masking condition (*F*_5,170_ = 22.69, *p* < 0.001, η_p_^2^ = 0.40). The interaction was not significant (*F*_10,170_ = 1.51, *p* = 0.139, η_p_^2^ = 0.082), suggesting that the broad tuning of masking was similar across groups.

A one-way ANOVA between the accuracy of AVGPs, NAVGPs and CONs in the no-masking condition revealed a significant effect of the group (*F*_2,34_ = 5.75, *p* = 0.007). Post-hoc comparisons using a false discovery rate (FDR) at 0.05^48^ revealed only a significant difference between CONs and NAVGPs in accuracy for the no-masking condition (*adjusted-p* = 0.006). The one-way ANOVA did not reveal any significant difference between AVGPs and NAVGPs (*adjusted-p* = 0.12) and between AVGPs and CONs (*adjusted-p* = 0.12).

In order to further assess the effect of the masking stimulus we conducted a separate repeated measures ANOVA for each group.

For the AVGPs group the ANOVA revealed a significant effect of the masking condition (*F*_5,55_ = 5.67, *p* = 0.0001, η_p_^2^ = 0.34). *Post-hoc* comparisons using FDR at 0.05 revealed that all the target-sample-mask direction differences were significantly different from the no-mask condition (*p* < 0.05). A one-way repeated-measures ANOVA performed only on the five target-sample-mask direction differences revealed no significant effect (*F*_4,44_ = 0.618, *p* = 0.65, η_p_^2^ = 0.053). Additionally, a one-way repeated-measures ANOVA with polynomial trend analysis did not discover any significant trend (linear, quadratic, cubic, quartic, all *p* > 0.05), suggesting negligible tuning of masking.

For the NAVGPs group the repeated measures ANOVA revealed a significant effect of the masking (*F*_5,45_ = 6.38, *p* = 0.0001, η_p_^2^ = 0.42). *Post-hoc* comparisons using FDR at 0.05 revealed that all the target-sample-mask direction differences were significantly different from the no-mask condition (*p* < 0.05). A one-way repeated-measures ANOVA performed on the five target-sample-mask direction differences revealed no significant effect (*F*_4,36_ = 1.49, *p* = 0.225, η_p_^2^ = 0.142). The trend analysis did not report any significant trend (all *p* > 0.05).

Similarly, for the CONs group the ANOVA revealed a significant effect of the masking (*F*_5,70_ = 13.97, *p* < 0.001, η_p_^2^ = 0.5). *Post-hoc* comparisons using FDR at 0.05, revealed that all the target sample-mask direction differences were significantly different from the no-mask condition (*p* < 0.001). A one-way repeated-measures ANOVA performed on the five target-sample-mask direction differences did not reveal any significant effect (*F*_4,56_ = 1.135, *p* = 0.349, η_p_^2^ = 0.075). Additionally, the trend analysis did not report any significant trend (all *p* > 0.05), again suggesting negligible tuning of masking. Therefore, we found that the masking stimulus affected task performance regardless of its motion direction relative to that of the sample. However, there is little or no tuning of masking.

Figure 2b shows the performance of AVGPs, NAVGPs and CONs in the no-mask and mask conditions, after pooling over all the sample-mask direction differences. On average, the intervening masking reduced performance by 8.31%, 9.7% and 16.85% in AVGPs, NAVGPs and CONs, respectively. A mixed ANOVA revealed a significant effect of the group (*F*_2,34_ = 8.93, *p* < 0.001, η_p_^2^ = 0.344), a significant effect of the masking condition (i.e., no-masking vs. masking) (*F*_1,34_ = 93.88, *p* < 0.001, η_p_^2^ = 0.734) and a significant interaction between masking condition and group (*F*_2,34_ = 4.07, *p* = 0.026, η_p_^2^ = 0.193). For the group, post-hoc comparisons using FDR at 0.05 reported a significant difference between AVGPs and CONs (*adjusted-p* = 0.018), between NAVGPs and CONs (*adjusted-p* = 0.0003), but not a significant difference between AVGPs and NAVGPs (*adjusted-p* = 0.136). For the condition x group interaction, post-hoc comparisons reported a significant difference between the no-masking condition and the mask condition for all the three groups (*adjusted-p* < 0.001). Additionally, for the no-masking condition we found a significant difference in performance only between NAVGPs and CONs (*adjusted-p* = 0.006), however, for the masking condition post-hoc comparisons revealed a significant difference between AVGPs and CONs (*adjusted-p* = 0.0045), between NAVGPs and CONs (*adjusted-p* = 0.0003), but no difference between AVGPs and NAVGPs (*adjusted-p* = 0.27), suggesting very similar performance in the no-masking condition for the video-gaming groups. Additionally, both AVGPs and NAVGPs seem to have similar tolerance to the intervening masking stimulus, affecting their motion discrimination performance to a lesser degree than in CONs.

In Fig. 2c it is evident that AVGPs and NAVGPs have higher accuracies than CONs, but all three groups show better performance in the no-mask condition (i.e., all data points fall above the equal-performance diagonal with one exception). This pattern of results was consistent across nine of the twelve AVGPs and for all the NAVGPs and CONs.

### Modelling visual short-term memory

**Figure 3:**
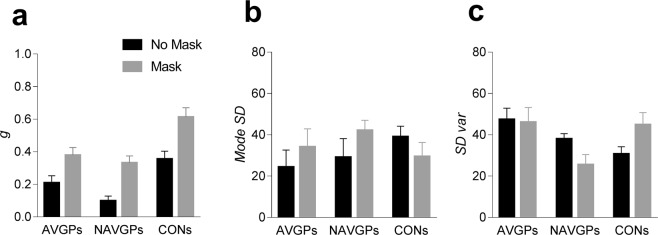
Parameters estimate of the variable precision model. (**a**) *g* (random guessing) for AVGPs, NAVGPs and CONs. (**b**) *Mode SD* (Precision) for the three groups. (**c**) *SD var* (Trial-to-trial precision variability) for the three groups. Error bars ± SEM.

In order to assess whether components of visual short-term memory differed across the three groups, accuracy data were fitted with a prominent model of visual short-term memory; the variable precision (VP) model^49–51^. Previous studies showed that visual short-term memory precision is continuous and variable across memory items and trials^49–52^, rather than continuous but equally distributed across items to be remembered^53^, or variable but deployed over a discrete number of memory slots^25^. Based on Zhang and Luck^25^, below the slot limit, memory items can be stored in more than one slot and averaged, thus producing high precision and high-resolution memory traces. However, beyond the slot limit, no information is stored resulting in an increase of guess rate (i.e., random responses). Therefore, the slot + averaging model of Zhang and Luck^25^ would predict that the errors in memory at recall emerge as a result of guessing and noise with which the item is stored. The VP model does not assume any memory slot limit, but resources are variable across trials and scale with the number of items to be remembered. Following the rationale of van den Berg *et al*.^50^, an observer should simultaneously memorize both the location and the motion direction of the two coherent RDKs. Each RDK is encoded with a certain precision. The VP model assumes that the observer’s internal measurement of a stimulus is noisy, and it follows a von Mises (circular normal) distribution, in which a high precision produces a narrower von Mises distribution. In the VP model, memory precision is variable across trials and the model assumes that it is drawn, independently across trials, from a gamma distribution. The model also assumes that mean precision (i.e., mean of the gamma distribution with a certain scale parameter), depends on set size in a power-law fashion (see Fig. 3a,b in van der Berg *et al*.^50^ for a schematic representation of the model), however, in our experiment we tested only one set size (i.e., two memory items). The VP model is characterized by three parameters: guess rate (*g*), mode SD and SD variability. The guess rate (*g*) expresses the probability with which the observer did not remember which of the two coherently moving patches in the test interval was drifting in a different direction with respect to the sample, and consequently guessed randomly. Mode SD represents the precision of the remembered items; high values in mode SD indicate a less precise memory representation. SD variability indicates trial-to-trial variation in memory precision; high values of SD variability indicate high trial-to-trial variability. We choose to fit the VP model instead of other available VSTM models also because of recent brain imaging evidence that shows how the superior intraparietal sulcus (sIPS) may be involved in the modulation of variability of memory precision^54^.

We fitted a two-alternative forced-choice (2AFC) version of the VP model using the Matlab MemToolbox^55^ (http://visionlab.github.io/MemToolbox/) in order to assess whether there are differences in the guessing rate (*g*), mode precision (*mode SD*) and SD variability (*SD var*) between the three groups. For each sample-mask direction difference we calculated the direction difference between the target sample and the relative test patch (i.e., the test patch occupying the same spatial location of the target sample) and we assigned the value 1 if the observer responded correctly or 0 otherwise. Based on Suchow *et al*.^55^, the model input was an array of target sample-test direction differences and an array of observer’s responses. The output of the model was an estimate of parameters *g*, *mode SD* and *SD var*. The MemToolbox uses Bayesian inference to derive a probability distribution over parameter values. This probability distribution describes the reasonableness of parameters after considering the observed data in light of a prior distribution (see Suchow *et al*.^55^ for a detailed description of model fitting and parameters estimation). The VP model was fitted to the entire set of each observer’s data. Since in the previous analysis we did not find any significant difference between the different masking directions used, the model was fitted just to the no-masking condition and to the masking condition, composed by all the sample-mask direction differences pooled.

### Results of the Variable Precision Model

Figure 3 shows the parameters estimate of the variable precision model. For guessing rate (*g*) (Fig. 3a), a mixed ANOVA revealed a significant effect of the group (*F*_2,34_ = 9.734, *p* < 0.001, η_p_^2^ = 0.364), a significant effect of the masking condition (*F*_1,34_ = 94.716, *p* < 0.001, η_p_^2^ = 0.735), but no significant interaction between group and masking condition (*F*_2,34_ = 1.465, *p* = 0.245, η_p_^2^ = 0.0793). For the group, post-hoc comparisons using FDR at 0.05 revealed a significant difference between AVGPs and CONs (*adjusted-p* = 0.006), between NAVGPs and CONs (*adjusted-p* = 0.0001), but no significant difference between AVGPs and NAVGPs (*adjusted-p* = 0.255), suggesting an overall higher guessing rate in CONs. A higher guessing rate was also evident in the masking condition compared to the no-masking condition (mean difference: 0.219, SE: 0.0223).

For mode precision (*Mode SD*) (Fig. 3b), a mixed ANOVA did not reveal any significant main effect or interaction (group: *F*_2,34_ = 0.386, *p* = 0.682, η_p_^2^ = 0.022; masking condition: *F*_1,34_ = 0.542, *p* = 0.467, η_p_^2^ = 0.0157; interaction group x masking condition: *F*_2,34_ = 1.517, *p* = 0.234, η_p_^2^ = 0.0819). Additionally, from Fig. 3b, there seems to be a linear trend for the no-masking condition, with mode SD linearly increasing for the three groups. A Jonckheere-Terpstra trend analysis did not reveal a significant trend for the order AVGPs, NAVGPs and CONs (*p* = 0.161).

For trial-to-trial precision variability (*SD var*) (Fig. 3c), a mixed ANOVA revealed no significant effect of the group (*F*_2,34_ = 2.675, *p* = 0.083, η_p_^2^ = 0.136) and masking condition (*F*_1,34_ = 0.001, *p* = 0.971, η_p_^2^ = 0.0001. However, the interaction masking condition x group was significant (*F*_2,34_ = 4.675, *p* = 0.016, η_p_^2^ = 0.216). For the interaction, post-hoc comparisons using FDR at 0.05 revealed a significant difference between AVGPs and CONs for the no-masking condition (*adjusted-p* = 0.021), but not a significant difference between AVGPs and NAVGPs (*adjusted-p* = 0.229) and between NAVGPs and CONs (*adjusted-p* = 0.237). Additionally, post-hoc comparisons also revealed a nearly significant difference between no-masking and masking conditions only for CONs (*adjusted-p* = 0.051).

In sum, fitting the accuracy data with the VP model^50^ revealed that the three groups do not differ in terms of precision of the memory representation (i.e., *Mode SD*). However, CONs exhibited a significantly higher guessing rate than video gamers and also higher trial-to-trial precision variability (*SD var*) than AVGPs. Additionally, a nearly significant difference between no-masking and masking conditions for *SD var* was found for CONs.

### Reaction Times

**Figure 4:**
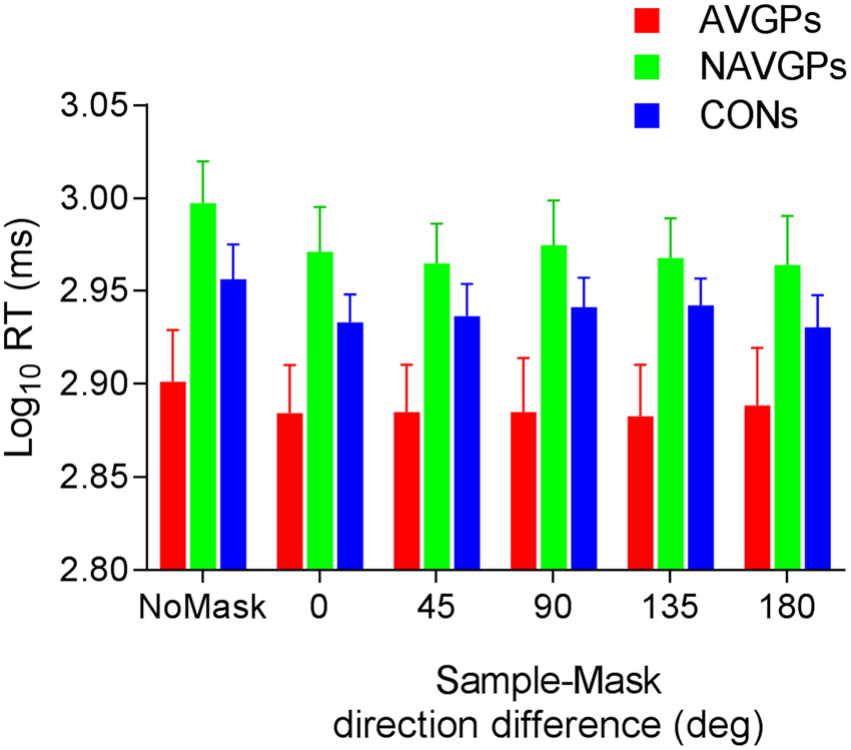
Reaction times in the visual short-term memory experiment. Filtered log_10_ reaction times (ms) of correct responses as a function of the direction difference between sample and mask. Results of the no-mask condition are plotted along the leftmost position of the abscissa. The colours signify the results from the three groups tested. Error bars ± SEM.

Figure 4 shows the log_10_ reaction times (RTs) for correct responses only. Before transforming RTs in log_10_, for each observer and condition, outlier RTs were filtered out following the procedure of Lachaud and Renaud^56^. Specifically, we calculated the median of the RTs, and trials in which RTs were ±3 *MAD* (median absolute deviation) or more from the median were removed from the analysis. A mixed ANOVA on log_10_(RT_s_) revealed a significant effect of the group (*F*_2,34_ = 3.587, *p* = 0.039, η_p_^2^ = 0.174), a significant effect of direction difference between sample and mask (*F*_3.376,114.78_ = 4.24, *p* = 0.005, η_p_^2^ = 0.111), but no interaction between sample-mask difference and group (*F*_10,170_ = 0.422, *p* = 0.934, η_p_^2^ = 0.024). When the sphericity assumption was violated, degrees of freedom were corrected using the Greenhouse-Geisser correction. For the group, post-hoc comparisons using FDR at 0.05 reported a significant difference between AVGPs and NAVGPs (*adjusted-p* = 0.039), but no difference between CONs and AVGPs (*adjusted-p* = 0.135) and between CONs and NAVGPs (*adjusted-p* = 0.276). With respect to the effect of direction difference between sample and mask, post-hoc comparisons using FDR at 0.05, revealed that reaction times in the no-masking condition were systematically greater than the reaction times of all the other sample-mask direction differences (all *adjusted-p* values < 0.01). Additionally, there were no significant differences in reaction times between the other sample-mask direction differences (all *adjusted-p* values > 0.05).

An additional analysis of variance for repeated measures with trend analysis on sample-mask direction differences revealed significant linear (*F*_1,34_ = 8.217, *p* = 0.007, *partial-η*^2^ = 0.195) and cubic (*F*_1,34_ = 12.74, *p* = 0.001, *partial-η*^2^ = 0.273) trends. Higher-order trends were not significant (Quadratic, *F*_1,34_ = 2.09, *p* = 0.157, *partial-η*^2^ = 0.058; quartic, *F*_1,34_ = 1.192, *p* = 0.283, *partial-η*^2^ = 0.034; quintic, *F*_1,34_ = 0.124, *p* = 0.727, *partial-η*^2^ = 0.004).

Additionally, a one-way ANOVA conducted between log_10_(RT_s_) of AVGPs, NAVGPs and CONs in the no-masking condition, revealed a significant effect of the group (*F*_2,34_ = 3.757, *p* = 0.034). Post-hoc comparisons using FDR at 0.05, reported a significant difference between AVGPs and NAVGPs (*adjusted-p* = 0.03), but no difference between AVGPs and CONs (*adjusted-p* = 0.151) and between NAVGPs and CONs (*adjusted-p* = 0.22). On average, CONs exhibit reaction times that fell in between those of AVGPs and NAVGPs. Additionally, response times of NAVGPs were longer than reaction times of AVGPs, but NAVGPs showed similar performance values to those of AVGPs. In order to better understand this speed-accuracy trade-off between the two video game groups, reaction times data were fitted with the standard diffusion decision model^57–59^.

### Diffusion model analysis

**Figure 5:**
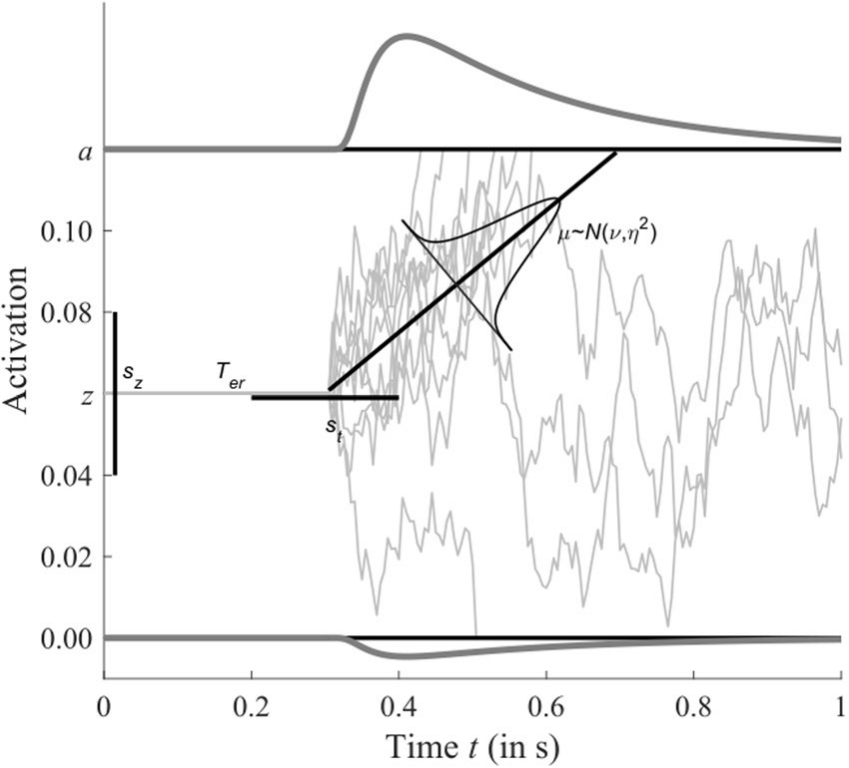
The diffusion model of response times. Decision latency is modelled as the first-passage time of a Wiener process with drift *µ*, starting at *z*, to one of two evidence thresholds, placed at *a* and 0. Due to random noise, the first-passage occurs at different times and at different criteria. The corresponding density functions of first passage is plotted at either criterion. In addition to the decision latency, there is a non-decision latency *T*_er_. In the full model, drift, starting point, and non-decision time are assumed to vary from trial-to-trial. The drift is assumed to be normally distributed with mean *v* and variance *η*^2^. Starting point and non-decision time are assumed to be uniformly distributed with distribution width *s*_z_ and *s*_t_, respectively.

For a combined analysis of response times and accuracy rates, we fitted the standard diffusion decision model^57–59^ to the data of all participants. The diffusion model is a model for perceptual decisions with two alternatives^60^. It is based on the assumption that decision times can be described by a Wiener diffusion process subject to two response criteria, representing evidence thresholds for each of the two alternatives. Due to random perturbations, a response criterion is reached sometimes sooner, sometimes later, and can also reach the criterion associated with an incorrect response (Fig. 5). Hence, the diffusion model allows for a complete analysis of RTs, including response accuracy and the distributions of both correct and incorrect responses.

We fitted the standard diffusion model with trial-to-trial variation in drift rates, starting point, and non-decision times to the RT distributions (i.e., the 10%, 30%, 50%, 70%, and 90% quantiles) of trials without masking and trials with masking between the sample and the test intervals. Since we did not find a significant effect of the sample-mask direction difference with respect to accuracies, RT data were pooled across all masking conditions.

The standard version of the diffusion model has seven free parameters: in addition to the barrier separation (*a*), drift rate (*v*), starting point (*z*), and non-decision time (*T*_er_), the model includes trial-by-trial variation in drift rate (*η*), starting point (*s*_z_), and non-decision time (*s*_t_). When fitting this model, we allowed for separate drift rates for decisions in trials with and without masking. A difference in drift rates — if present — would reflect an effect of the masking, reflecting how detrimental the mask had been to the memory representation of the sample stimulus. It turned out that fixing the starting point to *a*/2 (i.e., unbiased decision making) did not affect the model fits much, so we fixed this parameter and set its variation to zero. The whole model had six free parameters to explain RT distributions for correct and error RTs in two conditions (i.e., four distributions).

Predictions of the diffusion model were obtained using an efficient evaluation of the first-passage distribution with variable drift^61^ and numerical integration to account for variable non-decision time^62^. Parameter estimates were obtained by quantile maximum likelihood estimation^63^ using the aforementioned quantiles. The resulting estimates of the model were tested for group differences by means of a one-factorial ANOVA. Since this involved six statistical tests, one for each parameter, the results were corrected for multiple comparisons using FDR correction^48^. Only parameters that exhibited significant group differences after FDR correction at 0.05 were subsequently tested in single group comparisons by means of (two-tailed) *t*-tests.

### Results

**Figure 6:**
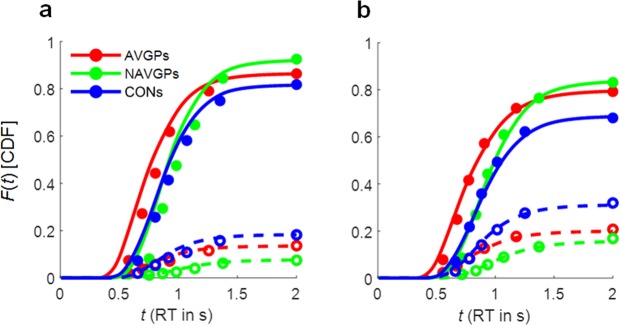
Empirical (10%, 30%, 50%/median, 70%, and 90% quantiles plus overall response probability, circles) and predicted (unmarked points connected with lines) cumulative distribution function of correct (solid circles and lines) and error responses (open circles and dashed lines). (**a**) Data and model predictions for the no-masking condition. (**b**) Data and model predictions for the (pooled) masking condition.

The parameter estimates of the model were in the usual range for a perceptual decision task (Table 1) and the overall model fit was acceptable (Fig. 6). The fit to RTs for the RT distributions in the masking condition was very good (Fig. 6b), whereas in the no-masking condition the fit was slightly worse (Fig. 6a). This lack of fit was detected during model selection, and it was found that an additional free parameter for the no-masking condition (i.e., a separate response criterion or non-decision time latency) increased the quality of the fit. The quantitative increment of the goodness-of-fit of the model was marginal, however, since the no-masking condition contained distinctly fewer trials than the masking condition. Therefore, we did not include a separate non-decision time latency for those trials. All diffusion model parameters except the drift rate are the same between conditions. Fixing the drift rate to be equal would lead to identical model predictions and, hence, considerably worse model fits (Fig. 6). In statistical terms, the drift rates between the two conditions are significantly different for AVGPs (*X*^2^ = 23.16, *df* = 12, *p* = 0.03) and CONs (*X*^2^ = 60.78, *df* = 15, *p* < 0.001), but not for NAVGPs (*X*^2^ = 16.05, *df* = 10, *p* = 0.10).

**Table 1:** Mean parameter estimates (±SE) and significance tests of the diffusion model fit.

Parameter	Group			*F*	*p*	*p*_adj_
	AVGPs	NAVGPs	CONs			
*a*	0.119 (±0.007)	0.127 (±0.008)	0.109 (±0.004)	2.202	0.126	0.463
*v*(mask)	0.157 (±0.016)	0.233 (±0.035)	0.099 (±0.016)	9.402	<0.001	0.008
*v*(no mask)	0.226 (±0.028)	0.338 (±0.048)	0.200 (±0.035)	3.626	0.037	0.183
*η*	0.088 (±0.024)	0.145 (±0.035)	0.097 (±0.023)	1.180	0.320	0.940
*T*_er_	0.562 (±0.035)	0.738 (±0.047)	0.683 (±0.033)	5.387	0.009	0.068
*s*_t_	0.388 (±0.056)	0.421 (±0.049)	0.419 (±0.055)	0.109	0.897	0.99

Note—Parameters are evidence threshold (*a*), (mean) drift rates (*v*), drift rate standard deviation (*η*), (mean) non-decision time (*T*_er_), and variation of non-decision time (*s*_t_). Starting point was fixed (*z* = *a*/2, *s*_z_ = 0). *F*- and *p*-values refer to results of group comparisons (ANOVA), the adjusted *p*-values (*p*_adj_) were obtained after FDR correction.

We obtained a significant group difference for the drift rate in masking trials (*F*_2,34_ = 9.40, *adjusted-p* = 0.008). There were also significant differences between the drift rate in no-masking trials and non-decision time latency, but these differences were no longer significant after FDR correction. Most importantly, we found no evidence for different response criteria (*a*) across the three groups (*F*_2,35_ = 2.20, *adjusted-p* = 0. 46), which speaks against a speed-accuracy trade-off as an explanation for the observed group differences in accuracy and RT. The drift rate in masking trials was significantly higher for AVGPs (*v*_mask_ = 0.16) than for CONs (*v*_mask_ = 0.1, *t*_25_ = 2.54, *p* = 0.018) and significantly higher for NAVGPs (*v*_mask_ = 0.23) than for CONs (*t*_23_ = 3.93, *p* = 0.001). There was also a significant difference between AVGPs and NAVGPs (*t*_20_ = –2.1, *p* = 0.049).

These results speak against a speed-accuracy trade-off explanation for the differences across groups. Instead, the differences seem to reflect the quality of the VSTM representation: in all three groups, the mask after the sample stimulus negatively affected the representation in VSTM, and this masking effect was most pronounced in the CONs. In this group, the VSTM representation was particularly susceptible to the mask. But even in the conditions without the mask, VSTM representation at the end of the 3.2 s period was better in video game players than non-video game players. The fact that AVGPs had the fastest responses is partly due to a lower non-decision time latency. While this effect is not significant after FDR correction, non-decision latency was on average 120 ms to 180 ms lower in the AVGP group. The reason for this lower latency is difficult to determine; non-decision processes comprise processes such as encoding of the test stimulus, retrieval of the sample stimulus from VSTM, and the motor response, amongst others. Any of these processes could have contributed to a faster non-decision latency.

A problem with applying the standard diffusion model in this situation is that is does not account for guessing due to a total loss of the VSTM representation like the variable precision model. To overcome this shortcoming, we fitted a diffusion model that takes into account that the decision depends on an intact representation in VSTM. If this representation is lost, the drift necessarily must be (close to) zero. We accounted for this by fitting a mixture diffusion model in which some trials are modelled by the standard model as described in the previous section, but other trials are described by a diffusion model with zero drift. With zero drift, the unbiased decision model can predict “guessing”, since reaching either criterion happens with probability ½. The probability of losing the VSTM representation must be estimated from data; this adds two free parameters to the model because this probability is presumably different between masking and no-masking conditions. Two more parameters (response criterion *a* and non-decision time *T*_er_) for the guessing trials were needed to obtain an acceptable fit. On the other hand, the drift rate was no longer different between mask and no-masking conditions, so we constrained them to be equal. The guessing diffusion model had 9 free parameters.

The fit of the diffusion model that accounts for loss of the representation in VSTM by assuming a zero drift decision is comparable to the standard diffusion model and the estimated parameters closely reflect those of the standard model. However, the drift rates are no longer significantly different among groups (*F*_2,34_ = 0.84, *adjusted-p = *0.99). Instead, the probability of losing the VSTM representation in the masking condition is significantly different across the three groups (*F*_2,34_ = 10.5, *adjusted-p* = 0.007). Post-hoc comparisons using FDR at 0.05 showed that AVGPs (*t*_25_ = –3.73, *p* = 0.001) and the NAVGPs (*t*_23_ = –3.25, *p* = 0.004) guessed significantly less than CONs. In other words, CONs had a significantly higher amount of trials in which the VSTM representation was lost. The two video gamer groups did not differ significantly (*t*_20_ = 0.50, *p* = 0.62). We found no such effect in the no-masking condition and none of the other parameters differed significantly across the groups. This includes the drift rate, suggesting that, if the representation in VSTM was intact, the quality of information available for the decision was about the same in all three groups, irrespective of gaming experience. This essentially supports conclusions drawn from the variable precision model (see above), however, using the diffusion model, response times are taken into account as well.

### Learning Curves

Previous studies^64^ have argued that the generalized benefits seen in action video game players might be because AVGPs have an enhanced ability with respect to ‘*learning to learn*’. That is, when faced with a novel task, AVGPs start out not different from CONs, but they are able to learn the relevant statistics of the task at a faster rate, leading to a more rapid improvement in performance, as a function of exposure to the task. However, in our study we did not find differences in performance values between AVGPs and NAVGPs across all the masking conditions tested, suggesting that, with respect to this type of memory task, non-action video game playing is associated with approximately the same benefits as is action video game playing.

In the current study, observers performed a total of 288 trials split in 12 blocks. In order to examine whether video game players were better than non-video game players from the outset or whether all video game players started at the same level, with the difference first emerging over the course of exposure to the task, as would be predicted by the learning to learn hypothesis^64^, the 12 blocks were grouped in bins of two blocks, leading to 6 bins in total. We will refer to the bins as learning sessions. Binning was necessary because in each block the number of conditions differed, therefore we had to pool at least two blocks in order to estimate how performance varied with exposure to the task. To increase statistical power of this comparison, data from the masking conditions were pooled, since we did not find a significant difference between performance values of the different masking directions. We also estimated learning curves for the no-masking condition.

**Figure 7:**
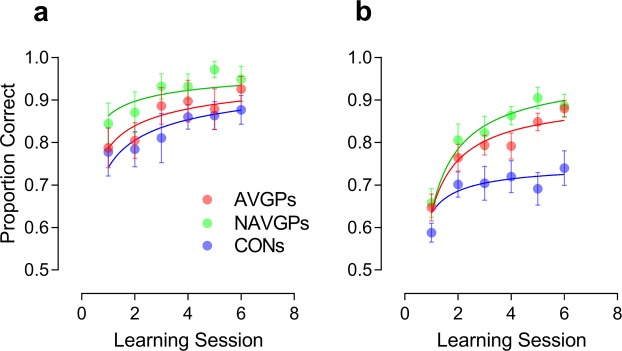
Learning curves for video game players and non-video game players with proportion of correct response as a function of the learning session. (**a**) No-masking condition. (**b**) Masking condition. Each learning session corresponds to a bin in which data from two consecutive experimental blocks were pooled. The curves represent an elaborated power function fitted to the performance values (see text for more details). Error bars ± SEM.

Figure 7 shows performance values as a function of the learning session. In order to test whether there were differences between learning rates of the three groups and whether video game players were better than non-video game players from the outset of the task, learning sessions were fitted with an elaborated power function of the form:

1 $$f(x)=a{x}^{-b}+c(1-{x}^{-b})$$

The function has been recast from the elaborated power function of Bejjanki *et al*.^64^. Parameter *a* indicates the accuracy in the first learning session, *b* is the learning rate (smaller values of *b* indicate slow improvements of the performance across learning sessions), and *c* indicates the asymptotic accuracy after an arbitrarily large number of learning sessions. Since parameters *a* and *c* indicate accuracies, they were constrained to vary between 0 and 1, whereas parameter *b* was constrained to assume only positive values in order to achieve strictly increasing learning curves.

The fitting procedure and model selection used was similar to that reported in Bejjanki *et al*.^64^ For the three groups, the fully saturated model consisted of nine parameters (one *a*, *b*, and *c* parameter per group). The maximally restricted model, i.e., the model that postulated no change between the groups, had only three parameters (*a*, *b*, and *c*), and assumed that the three parameters were the same across the groups (see Table 2). Between the fully saturated model and the maximally restricted model, a lattice of models with different numbers of parameters were fitted. The best-fitting model selection was based on the following *F*-test:

2 $$F=\frac{(RS{S}_{1}-RS{S}_{2})/(d{f}_{1}-d{f}_{2})}{RS{S}_{2}/d{f}_{2}}$$

where *RSS*_1_ and *df*_1_ are the residual sum of squares and the degrees of freedom of the simpler model, whereas *RSS*_2_ and *df*_2_ are the residual sum of squares and the degrees of freedom of the more complex model. The associated probability (*p*) can be computed as:

3 $$p=fcdf(F,d{f}_{1}-d{f}_{2},d{f}_{2})$$

where *fcdf* is the *F* cumulative distribution function^65^. If the resulting *p-value* was higher than the significance level (0.05), then the simpler model was likely to be correct. On the other hand, if the *p-value* was lower than the significance level, then the model with more parameters was likely to be correct. It should also be noted that the *F*-test can be used only with nested models (i.e., model A is nested in model B if parameters in model A are a subset of the parameters in model B). Comparisons between models with the same number of parameters (i.e., the same degrees of freedom) were performed using both the Akaike Information Criterion (AIC) test and the Bayesian Information Criterion (BIC). In this case the model with lower AIC and BIC is likely to be correct. Table 3 reports the lattice of models fitted to the performance values. All the possible pairs of models were compared without repetitions.

**Table 2:** Mean parameter estimates (±SE) and tests of the VSTM/diffusion model fit.

Parameter	Group			*F*	*p*	*p*_adj_
	AVGPs	NAVGPs	CONs			
*a*	0.128 (±0.013)	0.158 (±0.015)	0.134 (±0.012)	1.298	0.286	0.99
*v*	0.232 (±0.021)	0.279 (±0.022)	0.259 (±0.026)	0.844	0.439	0.99
*η*	0.045 (±0.037)	0.005 (±0.005)	0.015 (±0.008)	0.832	0.444	0.99
*T*_er_	0.554 (±0.046)	0.678 (±0.055)	0.702 (±0.050)	2.485	0.098	0.835
*s*_t_	0.339 (±0.061)	0.360 (±0.045)	0.391 (±0.054)	0.248	0.782	0.99
*p*_g_ (mask)	0.256 (±0.042)	0.284 (±0.036)	0.550 (±0.062)	10.50	<0.001	0.007
*p*_g_ (no mask)	0.081 (±0.038)	0.071 (±0.027)	0.233 (±0.052)	4.462	0.019	0.242
*a* (guess)	0.096 (±0.011)	0.116 (±0.005)	0.101 (±0.003)	1.973	0.155	0.984
*v* (guess)	(0)	(0)	(0)	—	—	—
*T*_er_ (guess)	0.681 (±0.092)	0.824 (±0.071)	0.736 (±0.050)	0.903	0.415	0.99

Note—Parameters are evidence threshold (*a*), (mean) drift rates (*v*), drift rate standard deviation (*η*), (mean) non-decision time (*T*_er_), and variation of non-decision time (*s*_t_). The guessing parameter (*p*_g_) refers to the percentage of trials in which the content in VSTM was lost. Parameters in parentheses are fixed. Starting point was fixed (*z* = *a*/2, *s*_z_ = 0). *F*- and *p*-values refer to results of group comparisons (ANOVA), the adjusted *p*-values (*p*_adj_) were obtained after FDR correction.

**Table 3:** Lattice of models fitted to the learning sessions of the three groups and for no-masking and masking conditions. *f*1(*x*) indicates the functions fitted to the AVGPs group, *f*2(*x*) indicates the functions fitted to the NAVGPs group and *f*3(*x*) the functions fitted to the CONs group.

Model name	Model function	Number of parameters
Fully Saturated	*f*1(*x*) = *a*1*x*^−*b*1^ + *c*1(1 − *x*^−*b*1^)	9
	*f*2(*x*) = *a*2*x*^−*b*2^ + *c*2(1 − *x*^−*b*2^)	
	*f*3(*x*) = *a*3*x*^−*b*3^ + *c*3(1 − *x*^−*b*3^)	
Restricted 1	*f*1(*x*) = *ax*^−*b*1^ + *c*1(1 − *x*^−*b*1^)	7
	*f*2(*x*) = *ax*^−*b*2^ + *c*2(1 − *x*^−*b*2^)	
	*f*3(*x*) = *ax*^−*b*3^ + *c*3(1 − *x*^−*b*3^)	
Restricted 2	*f*1(*x*) = *a*1*x*^−*b*^ + *c*1(1 − *x*^−*b*^)	7
	*f*2(*x*) = *a*2*x*^−*b*^ + *c*2(1 − *x*^−*b*^)	
	*f*3(*x*) = *a*3*x*^−*b*^ + *c*3(1 − *x*^−*b*^)	
Restricted 3	*f*1(*x*) = *a*1*x*^−*b*1^ + *c*(1 − *x*^−*b*1^)	7
	*f*2(*x*) = *a*2*x*^−*b*2^ + *c*(1 − *x*^−*b*2^)	
	*f*3(*x*) = *a*3*x*^−*b*3^ + *c*(1 − *x*^−*b*3^)	
Restricted 4	*f*1(*x*) = *a*1*x*^−*b*^ + *c*(1 − *x*^−*b*^)	5
	*f*2(*x*) = *a*2*x*^−*b*^ + *c*(1 − *x*^−*b*^)	
	*f*3(*x*) = *a*3*x*^−*b*^ + *c*(1 − *x*^−*b*^)	
Restricted 5	*f*1(*x*) = *a*1*x*^−*b*1^ + *c*(1 − *x*^−*b*1^)	5
	*f*2(*x*)=*ax*^−*b*2^ + *c*(1 − *x*^−*b*2^)	
	*f*3(*x*) = *a*3*x*^−*b*3^ + *c*(1 − *x*^−*b*3^)	
Restricted 6	*f*1(*x*) = *ax*^−*b*^ + *c*1(1 − *x*^−*b*^)	5
	*f*2(*x*) = *ax*^−*b*^ + *c*2(1 − *x*^−*b*^)	
	*f*3(*x*) = *ax*^−*b*1^ + *c*3(1 − *x*^−*b*^)	
Maximally Restricted	*f*(*x*) = *ax*^−*b*^ + *c*(1 − *x*^−*b*^)	3

For the no-masking condition (Fig. 7a) the best fitting model corresponded to the Restricted Model 4, i.e., the model with five parameters in which only the parameter *a* was varied across the three groups. As stated above a difference in parameter *a* indicates different accuracies between groups in the first learning session. The Restricted Model 4 had a global *R*^2^ = 0.851. The *a* parameter was 0.79 (SE: 0.02) for the AVGP group, 0.86 (SE: 0.02) for the NAVG group and 0.74 (SE: 0.02) for the CON group. The Restricted Model 4 was significantly better than the fully saturated model (i.e., the model with 9 parameters) (*F*_4,9_ = 1.17, *p* = 0.39), better than the maximally restricted model (*F*_2,13_ = 15.2, *p* < 0.001), and better than the model in which only parameter *b* (i.e., learning rate) varied across groups (i.e., Restricted model 5), with lower AIC and BIC (AIC Restricted model 4 = −117.43, AIC The Restricted Model 5 = −116.43; BIC Restricted Model 4 = −119.73, BIC Restricted Model 5 = −118.72). The Restricted Model 4 was also better than the model in which only the parameter *c* varied across groups (i.e., Restricted Model 6; AIC Restricted Model 6 = −113.35; BIC Restricted Model 6 = −115.64). The Restricted Model 4 was also better than all the models with 7 parameters (Restricted 4 vs. Restricted 1: *F*_2,11_ = 0.62, *p* = 0.56; Restricted 4 vs. Restricted Model 2: *F*_2,11_ = 2.8, *p* = 0.1; Restricted Model 4 vs. Restricted Model 3: *F*_2,11_ = 2.87, *p* = 0.1).

An additional analysis was performed in order to test whether parameter *a* differed between video gamers and between gamers and controls. In order to do this, the Restricted Model 4 was fitted separately to each dataset, but parameters *b* and *c* were fixed at 0.41 and 0.99, respectively. These values were taken from the previous *global* fit in which the same model (Restricted Model 4) was fitted to the three groups. Therefore, for the purpose of this analysis only the parameter *a* was free to vary and was constrained to assume values between 0 and 1.

When comparing the *a* parameter between AVGPs and NAVGPs the analysis reported that parameter *a* was different for each dataset (AIC same: −75.24, AIC different: −81.55). The same difference was found between AVGPs and CONs (AIC same: −82.13, AIC different: −83.57), and between NAVGPs and CONs (AIC same: −67.99, AIC different: −82.38). These results suggest that in the no-masking condition, the learning rate is similar across the three groups, and the three groups have the same asymptotic accuracy after an arbitrarily large number of learning sessions. However, video gamers performed better at the beginning of the experiment relative to non-gamers. Additionally, our analysis also pointed out that in the no-masking condition NAVGPs exhibited significantly higher initial accuracy rates than AVGPs.

For the masking condition (Fig. 7b) the best fitting model resulted to be Restricted Model 6, i.e., the model with five parameters in which only the *c* parameter was varied across groups. As stated above the *c* parameter indicates the asymptotic accuracy after an arbitrarily large number of learning sessions. The Restricted Model 6 had a global *R*^2^ = 0.946. The *c* parameter for the AVGP group was 0.91 (SE: 0.06), for the NAVG group was 0.97 (SE: 0.07) and for the CON group was 0.75 (SE: 0.03). The Restricted Model 6 and was significantly better than the fully saturated model (i.e., the model with 9 parameters) (*F*_4,9_ = 1.87, *p* = 0.2), better than the maximally restricted model (*F*_2,13_ = 49.58, *p* < 0.001), and better than the model in which only parameter *a* varied across groups (i.e., Restricted Model 4), with lower AIC and BIC (AIC Restricted Model 6 = −120.07, AIC Restricted Model 4 = −101.11; BIC Restricted Model 6 = −122.36, BIC Restricted Model 4 = −103.40). Restricted Model 6 was also better than the model in which only the parameter *b* (i.e., learning rate) varied across groups (i.e., Restricted Model 5; AIC Restricted Model 5 = −119.79; BIC Restricted Model 5 = −122.08). The Restricted Model 6 was also better than all the models with 7 parameters (Restricted Model 6 vs. Restricted Model 1: *F*_2,11_ = 3.31, *p* = 0.075; Restricted Model 6 vs. Restricted Model 2: *F*_2,11_ = 2.28, *p* = 0.15; Restricted Model 6 vs. Restricted Model 3: *F*_2,11_ = 1.42, *p* = 0.28).

An additional analysis was performed in order to test whether the asymptotic accuracy (i.e., parameter *c*) differed between video gamers and between gamers and controls. In order to do this, the Restricted Model 6 was fitted separately to each dataset, but parameters *a* and *b* were fixed at 0.63 and 0.85, respectively. These values were taken from the previous global fit in which the same model (Restricted Model 6) was fitted to the three groups. Therefore, for the purpose of this analysis only the parameter *c* was free to vary. When comparing the learning rate between AVGPs and NAVGPs the analysis reported that the asymptotic accuracy was different for each dataset (AIC same: −82.22, AIC different: −86.50). The same difference was found between AVGPs and CONs (AIC same: −64.70, AIC different: −82.53), and between NAVGPs and CONs (AIC same: −58.27, AIC different: −83.72). These results suggest that in the masking condition, the three groups had similar performance at the beginning of the experiment and also exhibited similar learning rates, but the asymptotic accuracy differed between the three groups, with video gamers exhibiting higher accuracy than controls after several learning sessions. Additionally, it seems that NAVGPs exhibited significantly higher asymptotic accuracy than both AVGPs and CONs.

## Discussion

In this study we investigated visual short-term memory (VSTM) in action video game players (AVGPs), non-action video game players (NAVGPs), and in non-video game playing controls (CONs). In particular, we used a visual masking paradigm previously used with macaque monkeys and humans. Participants were presented with a sample memory interval composed of two coherently moving RDKs and two randomly moving RDKs, each occupying a visual quadrant. Participants had to memorize both the spatial location and direction of the two coherently moving RDKs. After an interval of 3.2 s, a test display was presented and participants had to report which of the two coherently moving RDKs changed direction with respect to the sample display. In some of the trials, 0.2 s after the offset of the sample display, a masking stimulus was presented. The masking stimulus was composed by four coherently moving RKDs, and participants were instructed to ignore the intervening masking stimulus. We manipulated the motion direction of the masking RDK corresponding to the sample RDK that changed direction in the test interval (i.e., the target RDK). We measured both accuracy and response times.

The results showed that the intervening masking display presented shortly after the offset of the sample display interferes with performance showing a decrement in accuracy in mask compared to no-mask conditions. Though memory masking interfered with the performance of the three groups, the effects of masking were more pronounced for the non-video game playing controls (see Fig. 2). It should be noted that this result does not depend on group differences in their ability to discriminate the direction of the RDKs, since after initial training their performance was similar. However, the performance of the three groups significantly differed in the no-masking condition (Fig. 2a). The results showed a significant difference between CONs and NAVGPs in accuracy for the no-masking condition, but no significant difference between AVGPs and NAVGPs nor between AVGPs and CONs.

Overall, in the no-masking condition, video game players show a better performance than controls right at the outset of the experiment, as demonstrated by our analysis on learning sessions. Additionally, in the no-masking condition, we found that the learning rate and the asymptotic accuracy did not significantly differ across the three groups, indicating that the three groups differed in performance values at the outset of the experiment, subsequently converging with similar speed. On the other hand, for the masking condition, the three groups exhibited the same accuracy at the outset of the experiment and also the same learning rate, but the asymptotic accuracy differed amongst the groups. That is, video gamers could achieve better performance after a number of training sessions.

These results are only partially in agreement with those reported by Bejjanki *et al*.^64^. The authors claimed that AVGPs do not enter the perceptual task with improved perceptual templates, rather they found that the performance of AVGPs was indistinguishable from that of non-gaming controls at the beginning of the task. We confirmed these results only for the masking condition in which the best fitting model assumed the same starting accuracy. However, this does not hold for the no-masking condition in which the best fitting model assumed different starting accuracies, showing initially superior VSTM for video gamers than controls. Additionally, we could not confirm that video gamers learned more rapidly the statistics as they progressed on the task. This is because the best fitting models for the no-masking and masking conditions did not assume different learning rates across groups. On the other hand, in the masking condition, video game players outperformed controls showing significantly higher asymptotic accuracy after an arbitrarily large number of learning sessions, thus showing less interferences to intervening masking stimuli as they progressed on the task.

The reason behind the discrepancy in the present results and those of Bejjanki *et al*.^64^ might depend on the task used. In the case of Bejjanki *et al*.^64^, participants had to discriminate the orientation of a Gabor patch under different levels of external noise. It might be possible that for this type of low-level perceptual task, both video game players and non-gaming controls need time to learn the relevant statistics of the task to form a proper template of the target stimuli. The authors^64^ found that AVGPs are faster in learning statistics related to that perceptual task, as suggested by their perceptual training results. In our case, at least for the no-masking condition, it is possible that both action and non-action video game players had already an enhanced VSTM leading in self-selection and engagement in video game playing, though this advantage is lost when introducing the masking RDKs after the sample. In the no-masking condition it should also be noted that even though video game players were better than controls right at the outset of the experiment, they showed no faster improvement as the task progressed.

Additionally, when correct and error responses were fitted with the variable precision model^50^, the results revealed a difference in terms of guessing rate between groups, with CONs guessing more than video-game players, and exhibiting slightly higher trial-to-trail variability in memory precision than AVGPs, but not with respect to NAVGPs. The VSTM modelling results then suggest that the overall VSTM precision is similar across the three groups, but CONs made more random responses than video-game players.

In an analysis of the reaction times, we did not find any evidence for faster responses (i.e., lower reaction times) in AVGPs compared to CONs. This result is consistent with our previous work^3^ in which we did not find differences in terms of reaction times between AVGPs and CONs. However, our NAVGPs showed a different pattern of results. Though VSTM performance was very similar across AVGPs and NAVGPs, the reaction times of NAVGPs were much greater than those of AVGPs. On the other hand, we found intermediate response times for CONs. In order to better investigate this possible speed-accuracy trade-off between AVGPs and NAVGPs, reaction times data were analysed with the standard diffusion decision model^57–60^. According to the analysis of the diffusion model, the aforementioned RT differences between the three groups are mostly attributable to the latency of non-decision processes and do not reflect a speed-accuracy trade-off. This is further supported by the finding that the response criteria are approximately the same across the three groups. The non-gamers set the lowest (i.e., most liberal) criterion, on average, but not significantly lower than the video gamers. The same holds for the estimated non-decision time in the three groups who show the same ordering as the mean reaction times, but these differences were not statistically significant. The results of the diffusion model fits further suggest that persons who regularly play video games were less susceptible to interference by the mask. Indicative of the speed of information accumulation, the diffusion drift rates were higher for the video-gaming groups compared to the non-gamers. This trend was observed in both masking and no-masking conditions, whereas the effect was significant only in the masking condition. It seems unlikely that this difference is attributable to the test stimulus. Rather, this finding suggests that video gamers were more efficient in holding the sample stimulus in visual working memory until the test stimulus was presented. A similar conclusion can be drawn from the diffusion model that includes guessing due to loss of the VSTM representation. According to this model, the two groups of video gamers had to rely less on guessing because they more often had the information from VSTM available for the decision. In sum, the results of the diffusion model support the notion that video game players were more efficient in shielding the visual working memory trace of the sample stimulus from interference of the mask. Non-gaming controls, on the other hand, exhibited an overall poorer performance and higher guessing rates, as estimated both with the variable precision model^49–51^ and the diffusion model^57–60^. These results are in line with those reported by Green *et al*.^1^, in which they found that AVGPs exhibit a higher rate of information processing and that motor processing was similar between AVGPs and non-gaming controls. It is difficult to assess whether the deterioration of performance in the mask condition is better described by a total loss of VSTM information in some trials or by a degradation of VSTM information. The variable precision model and the diffusion model modified to account for guesses are in support of the former interpretation, whereas the standard diffusion model analysis supports the latter.

Another result that is worth mentioning is that in our previous study^34^ we found that masking interfered more when its direction matched that of the target sample. However, in the present study there is no difference in performance as a function of the sample-masking direction difference. The reason is not clear, but it might depend on our use of 100% coherent motion in the sample and mask displays, whereas in our earlier study^34^ we used globally moving RDKs with a signal-to-noise ratio producing approximately 84% correct performance in direction discrimination. Physiological and psychophysical evidence indicates that in motion area MT the directional tuning of neurones is largely invariant with motion coherence and contrast^66,67^.

In conclusion, we showed that both action and non-action video game playing is associated with an improvement in VSTM for moving objects leading to a visual memory representation that is more robust to external interference. Video game players also exhibit a lower guess rate than non-gaming controls, although memory precision and across trial precision variability were relatively similar across the three groups. Computational modelling of reaction times and accuracies pointed out additional advantages of AVGPs over both NAVGPs and CONs. AVGPs exhibit more efficient information processing and faster response execution than NAVGPs and CONs. Owing to the lack of random group assignment we cannot make any definitive statements concerning the cause and effect of the observed group differences. Further studies should determine the effects of repeated video game playing on VSTM task behavior in a longitudinal study approach with random assignment to the video gaming and control groups.

Why it is important to examine VSTM in video game players? As mentioned above, VSTM is involved in a number of important cognitive functions. Video game training and in particular training with action video games may represent a cost-effective and non-invasive training tool to improve VSTM in vulnerable populations. Action video game training can be used for example to counteract the normal VSTM decay in aging^26,27^, and even for rehabilitation purposes in patients with mild cognitive impairment^68^, patients with medial temporal lobe damage^69^, mild Alzheimer disease^70^ and Parkinson disease^71^. Beneficial effects of action video game training on VSTM could also be used to improve visually guided behaviour, with important implications in everyday tasks, driving or sport activities^19^.

## Supplementary information

Author_List_Changes_Approval_form_1550147288_15

## Data Availability

The analysed datasets are available from the corresponding author upon request.
