# Supplementary material for: Visual short-term memory for coherent motion in video game players: evidence from a memory-masking paradigm
Source: Sci Rep. 2019 Apr 15;9:6027. doi: 10.1038/s41598-019-42593-0 (PMC6465596; doi:10.1038/s41598-019-42593-0)
Supplement: Supplementary file 1 — Author_List_Changes_Approval_form_1550147288_15 [file 41598_2019_42593_MOESM1_ESM.docx]

In accordance to Nature Publishing Groups Authorship Policy we agree to change the authors of the manuscript as indicated below.

**NAME OF JOURNAL**: Scientific Reports

**TITLE OF MANUSCRIPT**: Visual short-term memory for coherent motion in video game players: evidence from a memory-masking paradigm

**MANUSCRIPT NUMBER**: SREP-18-07078B

**CORRESPONDING AUTHORS NAME: Andrea Pavan**

**PREVIOUS AUTHOR NAMES:**

Andrea Pavan, Martine Hobaek, Steven P. Blurton, Filippo Ghin, Mark W. Greenlee

**UPDATED AUTHOR NAMES:**

Andrea Pavan, Martine Hobaek, Steven P. Blurton, Adriano Contillo, Filippo Ghin, Mark W. Greenlee

**CHANGE TO AUTHOR LIST:** Adriano contillo has been added to the previous author list given his contribution with elaborated power function implementation, fitting procedure and model comparisons.

| **Print Name** | **Signature** | **Date** |
| --- | --- | --- |
| Andrea Pavan | 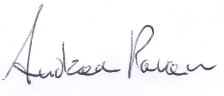 | 14/02/2019 |
| Martine Hobaek | 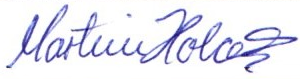 | 14/02/2019 |
| Steven P. Blurton | 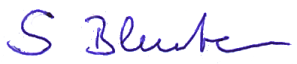 | 14/02/2019 |
| Adriano Contillo | 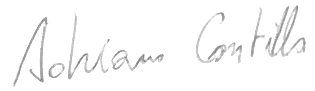 | 14/02/2019 |
| Filippo Ghin | 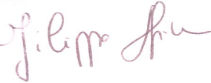 | 14/02/2019 |
| Mark W. Greenlee | 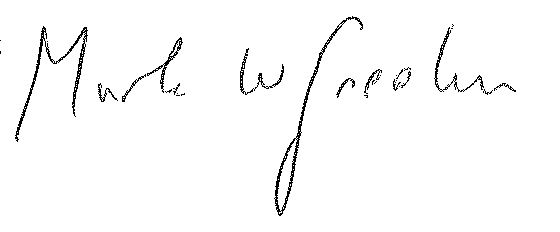 | 14/02/2019 |
